# Cancer drug development yesterday, today and tomorrow

**DOI:** 10.18632/oncoscience.583

**Published:** 2023-08-17

**Authors:** Elzbieta Izbicka, Robert T. Streeper

**Keywords:** cancer, drug development, chemotherapy targeted therapy

The “war on cancer” [1] began with the National Cancer Act, a United States federal law intended “to amend the Public Health Service Act so as to strengthen the National Cancer Institute in order to more effectively carry out the national effort against cancer” that was signed by President Richard Nixon on December 23, 1971. As the 50^th^ anniversary is now two years gone, the war has not been necessarily a blitzkrieg. To paraphrase Charles Dickens, today “it is the best of times, it is the worst of times” for cancer drugs. Great progress in cancer therapy has been made thanks to the combined impact of better supportive care, ever improving drugs and earlier cancer detection. On the other hand, the Anthropocene era brings new challenges due to increased human impact of environmental factors, which along with changes in diet and lifestyle may contribute to a worrisome increase in early-onset cancers, a situation viewed as a potential emerging global epidemic [2].

Conventional chemotherapy, one of the oldest anticancer weapons brought in late 1940s, encompasses alkylating agents, antimetabolites, natural products, hormones and steroids. Chemotherapy relies on the concept of eliminating the fast growing cancer cells [1] but adversely affects other fast growing normal cells in the body and lacks specificity. Despite these limitations, cytotoxic chemotherapy is widely used today.

Targeted therapies that emerged in the late 1980s are based on the assumption that cancer cells express and rely on the use of various unique targets for their survival. A success story of a targeted BCR-ABL tyrosine kinase inhibitor imatinib mesylate (Gleevec) spurred development of other targeted therapies, many of which effectively eradicated human cancers in animal models but failed to show clinical efficacy. Immunotherapy with monoclonal antibodies is a variation on the theme of targeted therapy. In particular, immune-checkpoint inhibitors do not kill cancer cells directly but instead mobilize the immune system to do the killing. In some cases, high success rates have been noted even in metastatic cancer [3] but substantial concerns remain regarding long-term toxicity [4]. Chimeric antigen receptor (CAR-T) targeted cell therapy has produced remarkable clinical responses with some hematological malignancies but is less effective in solid tumors and is limited by serious toxicities, antigen escape and restricted trafficking [5].

Targeted gene editing using CRISPR (clustered regularly interspaced palindromic repeat) with the bacterial RNA-guided CRISPR-Cas9 system has opened a new chapter in the drug development [6]. Several CRISPR-based products are undergoing clinical trials in patients with leukemias, lymphomas, solid tumors, and other diseases [7]. Whilst the bacterial CRISPR-Cas9 suffers from limited delivery to cells and tissues, a recent discovery of a CRISPR-like eukaryotic system based on the Fanzor protein, which uses RNA for precise DNA targeting, is expected to facilitate cell and tissue delivery for more efficient gene engineering [8].

Future cancer drug development might focus on a hybrid warfare that explores combinations of the old and new drugs for possible synergies, examines schedule and dose dependence of the treatments, and surveys individual proteomes and microbiomes for their potential as drug targets. The growth may be supplemented by combining and mining big data emerging from the laboratory and clinical research, and health records in various formats ranging from medical imaging to data from wearables and medical devices [9].

One can expect that artificial intelligence (AI) will play some role in the future drug development. A canary in the mine has already been spotted as the world’s first anti-fibrotic small molecule inhibitor discovered and designed using generative AI is being tested in the Phase II clinical trial [10].

Rewording Dr. Strangelove, does it mean that we must learn how to stop worrying and love the AI? Not necessarily so. The term AI could be a misnomer since we do not yet know what is actually the natural intelligence [11]. The limitations of AI stem from its reliance of on existing data, especially the quality, diversity and bias, regulatory challenges, and cost of infrastructure, to name a few. On the other hand, AI could be helpful in more routine tasks in target identification, new drug design and repurposing old drugs, virtual screening or lead optimization. One can anticipate peaceful coexistence with human creativity and knowledge leading and vetting all steps in drug discovery and development.
